# Case Report: Superior Mesenteric Artery Syndrome in an Adolescent With Cannabinoid Hyperemesis

**DOI:** 10.3389/fped.2022.830280

**Published:** 2022-02-21

**Authors:** Jonathan A. Berken, Samantha Saul, Peter T. Osgood

**Affiliations:** ^1^Division of Gastroenterology, Hepatology and Nutrition, Department of Pediatrics, Northwestern University Feinberg School of Medicine, Ann & Robert H. Lurie Children's Hospital of Chicago, Chicago, IL, United States; ^2^Northwestern University Feinberg School of Medicine, Chicago, IL, United States

**Keywords:** superior mesenteric artery syndrome, cannabinoid hyperemesis syndrome, Rome IV criteria, THC, case report

## Abstract

**Background:**

Superior mesenteric artery syndrome (SMAS) occurs when the third portion of the duodenum is compressed between the superior mesenteric artery (SMA) and the aorta, causing duodenal obstruction. This condition most commonly arises from marked weight loss that reduces the size of the fat pad between these vessels, causing greater acuity of angulation. We present an unusual case of SMAS occurring in an adolescent due to precipitous weight loss resulting from cannabinoid hyperemesis syndrome (CHS).

**Case Presentation:**

A 17-year-old adolescent presented emergently with voluminous bilious emesis. She endorsed a history of recent weight loss and a longstanding history of chronic heavy cannabis use associated with recurrent nausea and vomiting. Her chronic symptoms satisfied the Rome IV criteria for cannabinoid hyperemesis syndrome, but her acute vomiting symptoms were more extreme. Evaluation was significant for mild abdominal tenderness and fullness of the epigastrium. Contrast abdominal CT demonstrated moderate gastric and proximal duodenal distention with tapering of the lumen between the SMA and the aorta, consistent with SMAS.

**Conclusions:**

To our knowledge, this is the first reported case of SMAS occurring as the result of CHS. Clinicians should be aware of this possible juxtaposition, when a patient with a history of chronic excessive cannabis use, stereotypical vomiting resembling cyclic vomiting syndrome, and considerable rapid weight loss presents with a sudden exacerbation of symptoms, even when a normal BMI is maintained.

## Background

Superior mesenteric artery syndrome (SMAS) is a rare condition that occurs when the third portion of the duodenum is compressed between the superior mesenteric artery (SMA) and the aorta due to a narrowing of the angle between these vascular structures. The most common predisposing factor for the development of SMAS is significant weight loss leading to a diminution in retroperitoneal adipose tissue, including the fat pad between the SMA and the aorta, resulting in worsening acuity of angulation between these vessels and subsequent complete or partial obstruction of the duodenum. SMAS has been seen in association with conditions such as malignancy, malabsorption, trauma and burns, spinal cord injury, prolonged bedrest, and anorexia nervosa. Symptoms include postprandial epigastric pain, bilious emesis, and nausea (1, 2).

## Case Presentation

**Figure 1:**
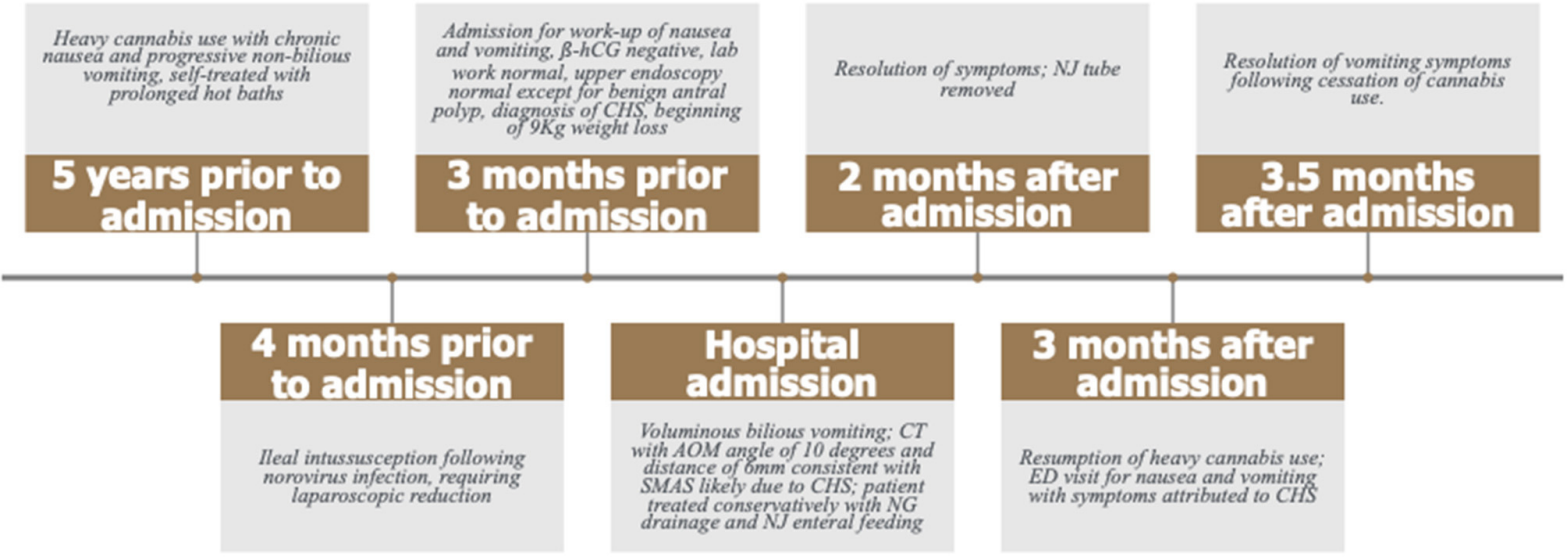
Timeline of the patient's clinical history.

A 17-year-old female presented to our emergency department (ED) with acute voluminous bilious emesis. Prior to this acute presentation, she had endorsed over 6 months of chronic nausea, especially upon awakening, progressing to protracted non-bilious emesis every 1–2 weeks. Symptoms were frequently relieved by prolonged hot baths. Her recurrent vomiting exacerbations were generally followed by 1 to 2 weeks of symptom reduction and self-imposed limitations in oral intake. Four months prior to her acute presentation, she had norovirus infection with mesenteric adenitis and mid-ileal small bowel-to-small bowel intussusception requiring laparoscopic reduction without resection. Evaluation of her chronic nausea and vomiting 3 months prior to admission revealed normal inflammatory, liver, and kidney markers, negative ß-hCG, and no evidence of sexually transmitted disease. An upper endoscopy performed at that time showed no anatomic abnormalities and was normal apart from a small hyperplastic antral polyp and microscopic non-eosinophilic esophagitis (Figure 1).

Her past medical history otherwise included hepatomegaly [seen on computed tomography (CT) scan several years prior to presentation, normal liver enzymes] and intermittent hyperglycemia, glucosuria, and ketonuria with a normal HgbA1c and no other clear clinical or laboratory evidence of diabetes. Of note, her prior CT scan noted no abnormalities in the proximal small bowel. Her social history was significant for over 5 years of heavy and accelerating cannabis use. At the time of presentation, she endorsed smoking 5–6 marijuana cigarettes daily for over 1 year, a habit she attributed to life stressors both at home and at school.

**Figure 2:**
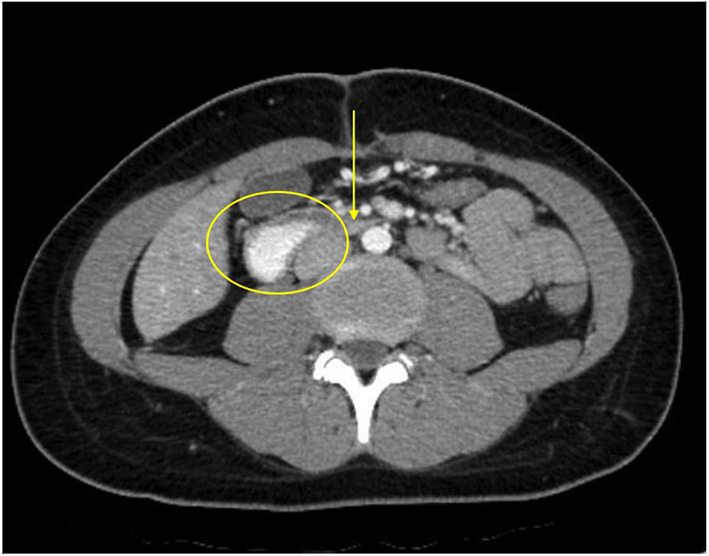
The patient's axial CT scan with oral contrast demonstrates the classic findings of superior mesenteric artery syndrome. The arrow points to the narrowed third segment of the duodenum, compressed between the superior mesenteric artery anteriorly and the abdominal aorta posteriorly. The duodenum proximal to the compressed segment is dilated and filled with contrast (circle).

**Figure 3:**
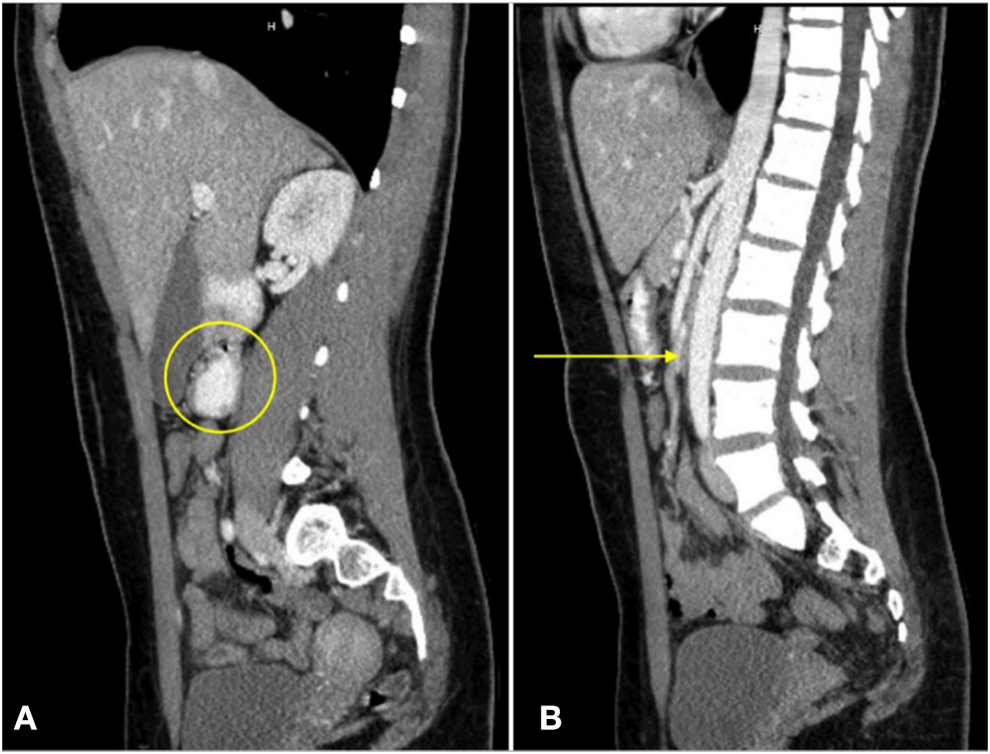
A sagittal CT view of the abdomen with oral contrast reveals dilation of the third portion of the duodenum [**(A)**, circle]. No enteral contrast is seen as the duodenum is compressed between the superior mesenteric artery and the abdominal aorta [**(B)**, arrow].

At presentation, the patient reported a rapid weight loss of more than 9 Kg. She denied constipation, diarrhea, or intentional vomiting or weight loss. She was noted to have a normal body mass index (BMI) of 23.46 Kg/m^2^, despite her weight loss. Physical examination was remarkable for diffuse abdominal tenderness and fullness to palpation, worst in the epigastrium. ß-hCG was again negative and urine toxicology screen was positive for tetrahydrocannabinol (THC). Abdominal x-ray was unremarkable. CT scan with contrast of the abdomen and pelvis revealed moderate gastric and proximal duodenal distention, with tapering of the duodenal lumen at the level of the SMA. The aortomesenteric (AOM) angle was 10 degrees and AOM distance was 6 mm. Taken together, these findings were suggestive of SMAS (Figures 2, 3) (3). There was no evidence of biliary or pancreatic pathology, adenopathy, or malignancy. A nasogastric tube was inserted for gastric and duodenal decompression and a nasojejunal (NJ) tube was placed to bypass the obstruction and provide nutrition and hydration.

While the patient's acute presentation and CT findings were attributable to SMAS, this did not explain her chronic antecedent symptoms of nausea, episodic vomiting, and weight loss. The patient's heavy chronic cannabis use, the longstanding nature of her complaints (>6 months), her otherwise reassuring evaluation, and her stereotypical pattern of episodic vomiting, satisfied the Rome IV criteria for cannabinoid hyperemesis syndrome (CHS), a well-defined disorder, characterized by prolonged cannabis use, cyclical nausea and vomiting with reduction or even complete cessation of symptoms between events, abdominal pain, and often compulsive hot water bathing for relief of symptoms (4). It was postulated that the weight loss she sustained as the result of frequent protracted CHS resulted in a diminution of the fat pad between the SMA and the aorta and eventual obstruction of the third portion of the duodenum leading to her acute presentation with voluminous bilious emesis. Acute symptoms improved with gastroduodenal decompression for several days. She was monitored for refeeding syndrome as NJ feedings were advanced to goal. The patient and her guardian demonstrated good understanding of her acute and chronic conditions and the goals for both her acute and long-term management. She did not follow-up with the Gastroenterology service but sought regular care with her primary care provider and was able to tolerate oral meals and have the NJ removed within 8 weeks of hospitalization. During this period, she was reportedly cannabis-free and had no further episodes of vomiting. Three months after admission, the patient presented again to the ED with intractable nausea and vomiting. She endorsed progressively escalating use of cannabis that approached the quantity and frequency at her prior admission. The resolution of symptoms during prolonged cessation of cannabis and the prompt resumption of symptoms with recurrent use further supported the diagnosis of CHS.

## Discussion And Conclusions

The patient's chronic illness satisfied the Rome IV criteria for CHS with stereotypical episodic vomiting resembling cyclic vomiting syndrome for >6 months, excessive cannabis use, and abatement of symptoms after prolonged cessation of cannabis use (4, 5). Her reliance on hot baths for relief of nausea and vomiting was supportive of CHS. While not all individuals with CHS experience significant weight loss, this patient's rapid loss following the progression of CHS precipitated the development of SMAS. While SMAS is most frequently seen in individuals with a low BMI, this case illustrates how an abnormally low BMI may not be necessary. In fact, a recent report describes the case of an adolescent male who, despite stable weight and BMI, acquired SMAS during a growth spurt whereby a gain of lean body mass occurred in tandem with a demonstrable loss of adipose tissue, including the fat pad between his SMA and aorta (6, 7).

The differential diagnosis for our patient's acute illness, in addition to SMA syndrome, included: small bowel obstruction, gastric or duodenal dysmotility (e.g., gastroparesis, ileus), collagen vascular disease (e.g., scleroderma), chronic idiopathic pseudo-obstruction, and peptic ulcer disease. The decreased AOM angle of less than 22 to 28 degrees and AOM distance between 2 and 8 mm in association with the patient's symptoms, however, were diagnostic of SMA syndrome (8–10).

The initial treatment for SMAS is conservative, with relief of the patient's obstructive symptoms through the passage of a nasogastric tube to decompress the stomach and proximal duodenum, followed by nutritional support with nasojejunal feeding distal to the site of obstruction, as in the present case (11). For those patients who can tolerate oral nutrition, frequent small feedings in the left lateral decubitus position are encouraged. Total parenteral nutrition may be required when enteral feeding of any sort is not tolerated (12). The goal of conservative therapy is to promote sufficient weight gain to increase the volume of the mesenteric fat pad and thus the AOM angle, alleviating bowel compression. When conservative measures to treat SMAS have failed, surgery may be indicated. Strong's procedure is the least invasive, as it does not involve bowel anastomosis. This surgical approach involves mobilizing the duodenum by dividing the ligament of Treitz and positioning the duodenum to the right of the SMA, allowing it to drop away from the aorta (13). Unfortunately, the procedure has a failure rate of up to 25 percent (13). Strong's procedure has been largely replaced by performing a duodenojejunostomy by dividing the fourth portion of the duodenum and creating a side to side anastomosis between the third portion of the duodenum and the end portion of the jejunum (14). A duodenojejunostomy restores bowel continuity and obviates the risk of blind loop syndrome. Both surgical procedures can now be performed laparoscopically (13, 14).

To our knowledge, this is the first reported case of SMAS occurring as the result of CHS. The patient's presentation with voluminous bilious vomiting in the setting of heavy marijuana use could have simply been attributed to exacerbation of her chronic CHS symptom pattern, leading to less comprehensive evaluation which might have missed or delayed the diagnosis of concurrent SMAS. Clinicians should be aware of the potential juxtaposition of cannabis hyperemesis and superior mesenteric artery syndromes in patients with a history of persistent cannabis use, stereotypical episodic vomiting resembling cyclic vomiting syndrome, and consequent weight loss, who present with a sudden increase in symptom severity or consistently bilious emesis.

## Data Availability Statement

The original contributions presented in the study are included in the article/supplementary material, further inquiries can be directed to the corresponding author/s.

## Author Contributions

JB: conceptualized, collected the radiographic images, drafted, reviewed, and revised the case report. SS and PO: interpreted radiographic images, revised, and critically reviewed the manuscript for important intellectual content. All authors approved the final manuscript as submitted and agree to be accountable for all aspects of the work.

## Conflict of Interest

The authors declare that the research was conducted in the absence of any commercial or financial relationships that could be construed as a potential conflict of interest.

## Publisher's Note

All claims expressed in this article are solely those of the authors and do not necessarily represent those of their affiliated organizations, or those of the publisher, the editors and the reviewers. Any product that may be evaluated in this article, or claim that may be made by its manufacturer, is not guaranteed or endorsed by the publisher.

## Abbreviations

SMA: superior mesenteric artery
SMAS: superior mesenteric artery syndrome
CHS: cannabinoid hyperemesis syndrome
CT: computerized tomography
BMI: body mass index
THC: tetrahydrocannabinol
AOM: aortomesenteric
NJ: nasojejunal.
